# Analysis of the Artistic Effect of Garden Plant Landscaping in Urban Greening

**DOI:** 10.1155/2022/2430067

**Published:** 2022-07-15

**Authors:** Lin Tian

**Affiliations:** College of Humanities and Arts, Chongqing University of Science and Technology, Chongqing 401331, China

## Abstract

At present, China is at an essential stage in the progress of social civilisation. At the same time, China's current economic level is developing rapidly and the level of urbanisation is also increasing. However, the uncontrolled development of urban space and excessive consumption of land resources have led to many urban ecological and environmental problems. As a result, there is an urgent need to improve the urban habitat. Plant landscaping is an important part of the urban environment. In addition, plant landscaping is also a crucial part of the visual arts and plays a key role in the shaping of urban spaces. In the context of the urban construction boom, people are increasingly demanding quality in the urban environment. Spatial scale, as a vital factor influencing the visual effect of planting, is gradually becoming a focus of landscape design. Urban greening based on plant landscape can not only improve the urban ecological environment and enhance people's quality of life but also resolve the contradiction between the demand for urban green space and the continuous reduction of urban greening land. Therefore, plant landscaping is recognised as a key step towards global sustainable development. However, according to current research and application practice, the development of urban greenery is limited and hindered by the value of plant landscaping and its applications. In addition, no comprehensive theoretical system has yet been established at the plant scale. In other words, there are still problems of scale in plant landscaping, such as unreasonable density, disproportion, and unclear hierarchy. Therefore, this paper begins with the definition of plant spatial scale in the city and analyses plant spatial types and scale characteristics from multiple perspectives, so as to establish an overall knowledge of plant landscaping. After that, through a study of the current situation of plant landscaping in cities, a quantitative analysis of the visual scale of plant space in urban squares is carried out to address the issues of plant scale design in this research. The focus of the analysis is on the scale of plants in relation to related elements. Furthermore, this study explores the influence of visual aesthetic scale and psychological scale on planting based on the functional scale of plant elements. Finally, the above quantitative analyses are applied to derive some relative data criteria and to summarise the design strategies for the spatial scale of planting in the city. The findings of this study can provide some guiding suggestions and references for the future construction of urban greenery, thus promoting the orderly development of plant landscaping in cities.

## 1. Introduction

As urbanisation continues to accelerate, it brings with it environmental issues such as rapid growth in urban population, expansion of spatial demand, and a diminishing amount of green space [1]. The development of cities requires a richer form of urban space organisation, leading to a boom in the construction of urban squares. As an important part of the urban green space system, the urban square is a gathering place for the public to release their emotions and relax [2]. Furthermore, it is considered to be an effective way to improve the urban environment [3]. As such, it has become a key part of urban image building. The planting of urban squares is one of the ways to showcase their great looks [4]. To be specific, comfortable and pleasant plant spaces are the aspiration of the general public and the relentless pursuit of landscape architects. Since the experience of plant space is largely influenced by scale design, the scale design of plant landscaping has become a hot topic of research for experts and scholars in recent years [5, 6]. The landscape is the cascade of spatial individuals that exist in the land area. At the same time, based on the cascade, the landscape can integrate space and space, object and object, object and space.

In the mid-18th century, the industrial revolution in Europe gave rise to a number of urban environmental problems [7]. In particular, the original ecological environment was severely damaged for the sake of urban development [8]. Against this background, people began to rethink the role of plants in urban landscaping and attempted to elaborate on the ecological, artistic, and humanistic perspectives of botanical landscaping. This has led to an important breakthrough in the development of the theory and practice of botanical landscaping [9]. The introduction and development of the concept of botanical landscaping in China began in the late 1980s. Experts and policymaking departments put forward the direction of ecological garden construction in response to the phenomenon that there were many unnatural hard landscapes such as buildings, rockeries, and fountains in garden construction at that time [10, 11]. Through the continuous in-depth research of a large number of scholars, the modern concept of botanical landscaping has been optimised to complement the traditional concept of botanical landscaping [12]. It is now generally accepted that the creation of a landscape is not only about plant material but also about ecology, biodiversity, and sustainable development [13]. In other words, contemporary botanical landscaping should focus not only on the rational matching of plants but also on the ecological benefits of botanical landscape construction [14]. After all, with the development of the construction industry, many relevant technologies have matured [15, 16]. In short, the spatial scale of plant landscaping has two dimensions. On the one hand, it refers to the scale of the surfaces that enclose plant space, which is the basic scale attribute of plant space and has a strong materialistic character [17]. On the other hand, there is a psychological scale of existence within the spatial scale of plants [18]. By controlling the relevant scale variables, the psychological impact of different spatial scales can be changed [19].

Scale is a specific property of space, and the plant space scale is a three-dimensional scale. The spatial scale of plants can be divided into dynamic and static scales according to the static and dynamic states of the space [20]. The dynamic scale mainly considers the visual effect of the planting as people move through the space [21]. Especially in the circulation space of the square, the dynamic scale provides a linear space for the public to walk through. Static scale refers mainly to the leisure space within the square [22]. As a result, the static scale requires the plant landscaping to consider that people have a great viewing point and to create a certain amount of privacy. The spatial scale is divided into three main stages according to the historical and empirical perspectives. The first one is the empirical scale stage, which is mainly reflected in the functional scale of space [23]. Through the exploration of natural space, people have gradually developed a scale of experience to help them understand the nature of space. The second stage is the scientific scale stage, which mainly reflects the scientific nature of spatial scale [24]. After New China, the pace of garden construction accelerated, and many scholars wanted to regulate the spatial sequence of the site through the design of spatial scale. Finally, spatial scale design entered a new stage of pursuing functional utility and visual enjoyment, reflecting the aesthetic nature of spatial scale [25]. Today, plant landscaping is no longer simply a softening agent for the hard landscape and an architectural appendage but a major component of the independent creation of spatial interfaces and visual landscapes. By combining with the topography, natural rocks, and surrounding architecture, plant landscaping can enhance artistic expression through unique modelling methods, forming a form of constructive art based on natural elements [26]. As a result, plant landscaping can create different visual effects from different perspectives, thus becoming a new way to beautify urban spaces.

At the beginning, China's gardens were basically civilian. Even the memorial gardens were modelled on pleasure gardens, which were in line with the humanistic requirements of the time [27]. The starting point and the scale of development of the gardens directly reflected the needs of the people's lives, especially their spiritual lives. Most of the gardens established at the time were enclosed complexes with captive zoos and some simple amusement facilities. This form was in keeping with the low material and cultural standard of living of the time. As the economy, culture, art, and modern technology developed, people were no longer satisfied with simple amusement [28]. In addition to this, people were also looking for more spiritual needs. As a result, different forms of gardens have emerged. These forms of gardening are in line with the current values of returning to nature and ecological effects [29]. In fact, modern gardens and social development are mutually influential. While social economy, politics, and culture determine the form and connotation of garden development, gardens also become part of social and economic activities, in turn promoting economic and cultural development and the improvement of material and spiritual civilisation. At present, many regions in China are using the development of gardens as a precursor to changing the face of the city and improving the investment environment, and this has achieved great results [30]. The economy and culture have led to the development of gardening, and the beautiful environment has brought about economic and cultural prosperity.

Although plant landscaping of many urban public spaces is constantly evolving, there are still many problems in the process of creating them. Planting a single variety of plants can not cope with the needs of seasonal changes, and plants suitable for different seasons should be planted in different seasons to play the role of landscape decoration [31]. Due to climatic constraints, many cities have few plant resources. At the same time, traditional perceptions of the ornamental value of plants have a clear bias. As a result, this has led to a uniform plant landscape from place to place. Secondly, there is a lack of aesthetics in the form of landscaping, a confusing hierarchy of plants, and a poor sense of spatial experience. In addition, some designers have chosen to use a large number of brightly coloured plants in their plant landscapes without following certain principles of formal beauty [32]. This results in a confusion of colour and form in the plant hierarchy. As a result, even if the plant material itself is good, the result is a disorderly situation. Thirdly, ecological benefits are ignored. As market competition intensifies, many real estate developers devote a lot of resources to the construction of the external environment of residential areas in order to meet the short-term needs of the market. Worse still, they use a large number of seasonal flowering plants in pursuit of a colourful landscape effect, without considering the actual growth of these plants, but focusing more on the rarity of their materials and neglecting the use of native plants. These phenomena not only fail to meet the functional and aesthetic needs of residents but also run counter to the concepts of resource conservation and sustainable development advocated by China. Therefore, an in-depth study and exploration of planting in urban gardens will not only meet the needs of residents in terms of use and aesthetics but also make full use of public space and avoid wasting resources.

The pursuit of a balanced relationship between man and nature is one of the main themes of social development today. Especially in economically prosperous cities, the desire and need for a green natural environment is becoming more and more evident. After all, the sustainable development of the urban ecological environment occupies an irreplaceable place in the development of human society. The area of urban greenery is an important criterion for assessing the quality of urban life and the quality of the environment and is an important part of urban planning. The current contradiction between the demand for urban green space and the continuous reduction of urban green space is becoming more and more serious. In order to improve this contradiction, it is necessary to focus on the development and use of urban garden space. To be specific, the application of three-dimensional urban space for greening and the development of a comprehensive range of plant landscaping is required. In addition, a variety of greening applications should be developed in order to enhance the quality of urban green space and improve the urban living environment without affecting the development of urbanisation.

## 2. Plant Landscaping

Through artistic techniques, plant landscaping can give full play to the natural beauty of the plants themselves in terms of form, line, and colour. At the same time, botanical landscaping can use trees, shrubs, vines, and herbs to create a beautiful and moving picture for people to enjoy. Firstly, planting can use plant materials such as trees, shrubs, vines, and groundcovers, which can be combined with other garden elements in different settings to create a beautiful landscape with both biological and aesthetic values. Secondly, planting elements can be combined with other elements of the garden. Then, based on the growth habits of the plants, a rational composition is used to create a unique landscape space that enriches people's needs. In addition to this, the concept of botanical landscaping is the use of plant material to create space. Specifically, while paying attention to compositional forms, colours, and other artistic techniques, reference must be made to their growth patterns and habits, and the scientific and rational composition of plant groups must be emphasised. Planting must therefore be a combination of aesthetic value and ecological benefit.

### 2.1. Development Status of Plant Landscaping

**Figure 1:**
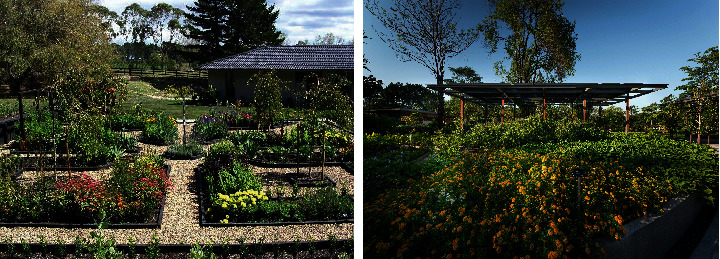
Plant landscaping in urban green-based gardens.

For northern cities in China, the number of residential areas has almost reached saturation and the pace of new developments is slowing down significantly. In this context, the renovation of planting will become a new direction for the future development of planting in cities. This means that instead of a new design for a large area, local improvements and enhancements will be made to certain areas of the residential landscape that need improvement. The Japanese have a more mature approach to the creation of public spaces. They do not hastily apply seedlings from plant catalogues to their landscapes. Instead, they learn about the colour, form, and ease of transplanting, the appropriate time of year for transplanting, and the hardiness, heat tolerance, and shade and sun preferences of the plants. This is followed by an in-depth analysis of the structure of the plant space to create a stunning plant landscape. For example, plant landscaping in urban green-based gardens is illustrated in Figure 1.

**Figure 2:**
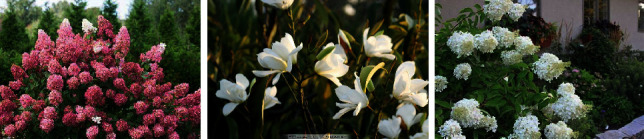
Various flowers in plant landscaping.

In fact, domestic landscaping is also developing rapidly. In contrast to the planting of residential areas, the planting of courtyards is more distinctive. In other words, there is a greater variety of plant species and a new expression of the overall colour palette and seasonal changes.  Figure 2 shows the planting of a courtyard. It uses a large number of perennial flowers instead of single seasonal flowers and lawns, such as cone hydrangea and White Jasmine for shrubs and jasmine and purple sage for groundcovers. This enriches the colours and layers of the plant space jasmine and gives the whole space a sense of wildness.

### 2.2. Function of Plant Landscaping

**Figure 3:**
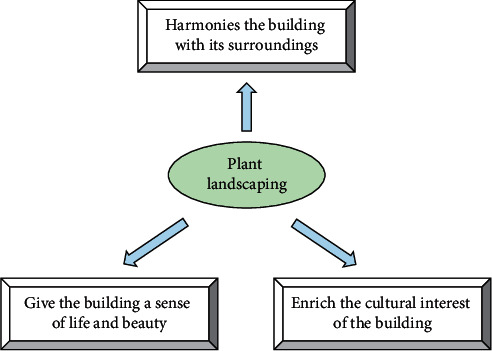
Functions of plant landscaping.

The landscape space varies according to the type of plant. The different forms and colours of the branches, flowers, leaves, and fruits of the plants form distinctive plant spaces. In addition to their own variation in volume, plants also have a seasonal variation in sequence. This process of change can be used for ornamental purposes. Since ancient times, it has been the custom in China to enjoy the seasonal beauty of plants. Therefore, the use of plants in urban public spaces has the following functions, as shown in Figure 3.

First of all, plant landscaping can harmonize buildings with their surroundings. In the city, many buildings have hard contours and a monochromatic palette. In contrast, plant material has a much softer texture and is rich in colour. The use of plants can therefore compensate for the lack of architecture, so that the rigid lines of the building are softened by the enclosure of plants. Careful planning of the composition, colour, and form of plants in plant landscaping will optimise the urban environment. In particular, it not only harmonizes the environment of public spaces in the city but also highlights important parts of them and forms the visual centre. For example, the visual effect of planting space can harmonize the size and proportion of residential spaces. In addition, the combination of buildings and plants can expand narrow spaces by creating space.

In addition, plant landscaping can give a sense of life to a building. To be specific, plants can change with the seasons to reveal their colourful and varied character. Buildings and walls with different styles can be decorated with different colours of plants. Plants with different flowering periods can be combined and configured according to their seasonal characteristics, thus giving public spaces a different view in different seasons.

Furthermore, plant landscaping can enrich the cultural interest of buildings. Imaginative plant communities can highlight the different landscape features of each space and thus enrich the urban landscape. As a result, all types of buildings can reflect their unique moods through appropriate planting. In other words, a reasonable configuration of plants around a building plays a pivotal role in creating a mood. People can resonate with the landscape, trigger associations, feel the feelings outside the scenery, and appreciate the beauty of plants while cultivating their sentiments.

### 2.3. Ecological Principle of Plant Landscaping

The global distribution of plants shows that temperature is an essential ecological factor affecting plant growth. This is mainly due to the fact that temperature directly influences photosynthesis, transpiration, and respiration, which in turn affect the growth and survival of plants. For most plants, either too high or too low temperature is not conducive to normal growth and development. As shown in Table 1, plants can be classified as hardy, semi-hardy, or intolerant according to their ability to adapt to temperature.

By judging the physiological changes in plants in response to changes in temperature, it is possible to manage the seasonal changes in plants in plant landscaping. As a result, the application of temperature in plant landscaping can be used to maximize the ornamental value of plants.

The effect of light on plants can be broadly expressed as the effect of light intensity on plants and the effect of light duration on plants. Depending on their light intensity requirements, plants can be classified as sunny, medium, or shady. The differences in light intensity are most pronounced in vertical spaces. Therefore, in planting, sunny plants, which require more light intensity, are suitable for the upper woods. Mesophotic plants are suitable for the midwoofs, while shady plants with a lower light demand are more appropriate for the lower woods (Table 2). The effect of light duration on plant growth is mainly in the form of growth promotion and inhibition. Generally speaking, increasing the duration of light promotes plant growth and, conversely, inhibits it. Therefore, this characteristic of plants can be used to domesticate exotic species in landscaping to create beautiful landscapes.

## 3. Spatial Scale of Plant Landscaping

The spatial scale of a planted landscape is defined as the proportional relationship between the planted landscape and people and the environment. Perceived through the human visual system, spatial scale is governed by a variety of factors, including physiological, psychological, environmental, and regional cultural influences. The study of scale in planted landscapes focuses more on the relationship with the proportions of the constituent elements than on the specific scale of the plants. In other words, the spatial scale of plants can be considered in a three-dimensional context, considering the principles of human vision and the surrounding environment. The final scale is a subjective perception of plant space, and, although there are commonalities, there is no single standard of scale.

### 3.1. Attribute of Spatial Scale

**Figure 4:**
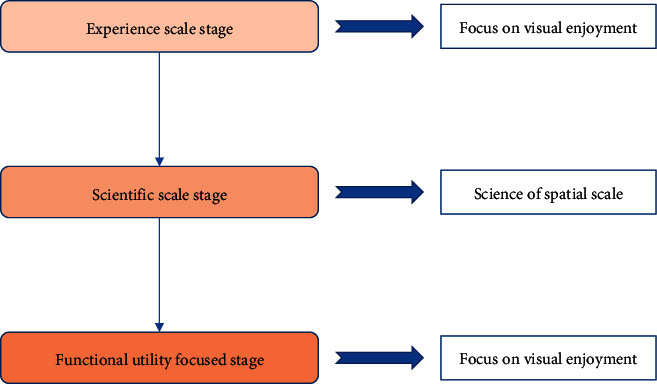
Process of spatial scale.

Attribute scale is a specific attribute of space, and the spatial scale of planting is a three-dimensional scale. The size and scale of planting affects the scale of public space and is the most important factor in forming different types of space. For example, medium or large tree trunks and canopies can form the vertical and top planes of a public space, thus controlling the size of the space. Small trees and some tall shrubs, on the other hand, form enclosures in the vertical plane or use linking techniques to optimise the spatial pattern. Small shrubs and groundcovers, however, often provide a rich ground space with a variety of colours and textures. As a result, the combination of plants of varying sizes and proportions can be unified in a public space to create different types of planting. The spatial scale can be divided into three main stages, as shown in Figure 4.

### 3.2. Open Space and Enclosed Space

**Figure 5:**
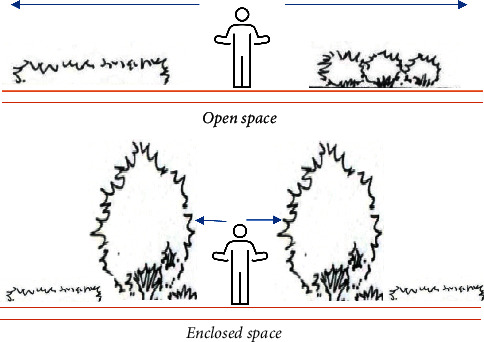
(a) Open and (b) enclosed spaces.

Open spaces are spaces where ground cover plants are used as a dividing element, creating a flowing and open visual effect. As a result, open spaces have less of a sense of spatial enclosure, with planting scales averaging below 1.3 m at eye level, and do not create a view block. Semi-open spaces, with taller vegetation shading one side of the open space, create a distinctly directional and extended one-way space. The semi-open space can therefore be used to direct the view and emphasise the main view. Semi-open spaces avoid close proximity to people, with the height of the cover being greater than 45° to the human eye, and the space is enclosed by greenery for a better visual effect. Enclosed spaces are urban plaza plant spaces where the top surface of the space is enclosed by plant material. This configuration allows the view to be blocked and provides a high degree of privacy. In summary, Figure 5 illustrates the open and enclosed spaces.

### 3.3. Circulation Space

**Figure 6:**
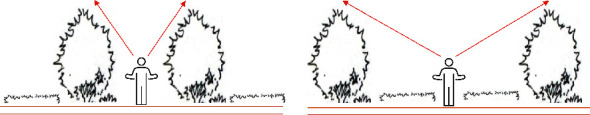
Circulation space.

A circulation space (Figure 6) is a linear space formed by a street tree as the basic enclosure element. Due to plant habits, circulation space acts as a link between spaces and divides them. As it is human nature to rely on nature, the use of plants as a spatial separator is the most human-friendly design approach. Street trees are considered the main medium for separating and linking the square landscape with the surrounding buildings, and the different distances between street trees give a different sense of spatial scale. As the seedling industry continues to develop and mature, a standardised and systematic system of seedling cultivation has been established and the variety of street trees is becoming more and more abundant. The development of the seedling industry has therefore contributed to the development of planted landscapes, which can meet more needs in landscape design and create a richer effect.

### 3.4. Objective Factors Affecting Spatial Scale of Plant Landscaping

The growth and development of plants is influenced by a variety of natural factors. Plants have vital signs and change over time and with the seasons. Therefore, knowledge of plant growth in different environments is the basis for plant space design. Only when plants are adapted to their environment can they grow and develop normally and form a stable community. The objective factors affecting spatial scale of plant landscaping are stated as follows.

Geographical culture can have an impact on the scale of plant space. Specifically, different regions have different scale symbols. In urban landscape design, plant spaces can be given a unique regional mood by changing the form of plant groups. Thus, spatial scales can be created secondarily to convey people's good intentions and achieve the atmosphere of an ideal plant space.

**Figure 7:**
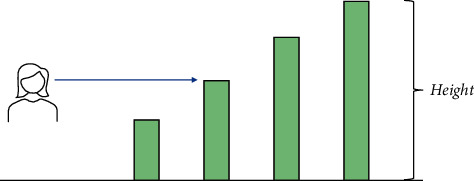
Influence of plant heights on sight.

In addition, human activities have an impact on the spatial scale of plants. People are both the implementers and the maintainers of plant space. Whether the arrangement of plant spaces is scientific and rational, and whether it is artistic, is one of the criteria for success or failure in plant space design. The maintenance and management of plants, such as pruning, fertilizing, irrigation, and weeding, also have an impact on the morphological dimensions of the plants and have a decisive influence on the stability of the plant space and the lasting effectiveness of the plants. Also, the different heights of the plants can have different effects on the line of sight, as can be seen in Figure 7.

## 4. Conclusion

Based on a general study of planting in public spaces in cities, this study summarises and analyses the current problems in planting and discusses the overall situation of the planted landscape. In addition, the spatial and scaling characteristics of urban planting, the current application of spatial scale, and the impact of related fields are analysed. The visual scale of urban plant space is then quantified and detailed from three perspectives: the configuration of plants, the relationship between the plant landscape and people, and the scale of plants and their surroundings. Finally, a design strategy for urban planting at spatial scale is attempted, laying a solid foundation for research into urban planting at spatial scale.

However, although there is no single correct description of the spatial scale of urban planting, it is still important to regulate the visual effect of urban space and is necessary to promote the spatial quality of planting in urban squares.

## Figures and Tables

**Table 1:** Different plant classification.

Plant classification	Geographical distribution	Representative plant
Hardy plant	Boreal and temperate	Moss and oleander
Semi-hardy plant	Subtropical and temperate	Balsam fir
Intolerant plant	Tropical	Rubber and coconut trees

**Table 2:** Light and the spatial distribution of plants.

Plant classification	Spatial distribution	Representative plant
Sunny plant	Upper layer of the forest	Moonflower and ginkgo trees
Medium plant	Lower and middle layers of the forest	Ivy
Shady plant	Surface	Mossy plants

## Data Availability

The labeled dataset used to support the findings of this study is available from the corresponding author upon request.
